# Supplementary material for: Bladder Cancer Immunotherapy by BCG Is Associated with a Significantly Reduced Risk of Alzheimer’s Disease and Parkinson’s Disease
Source: Vaccines (Basel). 2021 May 11;9(5):491. doi: 10.3390/vaccines9050491 (PMC8151667; doi:10.3390/vaccines9050491)
Supplement: Supplementary file 1 [file vaccines-09-00491-s001.zip › vaccine-code.pdf]

Sample code used to analyze the HUH cohort

This is a technical appendix of the article "Bladder Cancer Immunotherapy by BCG is associated with a significantly reduced risk of Alzheimer's disease and Parkinson's disease" by Klinger, et.al.

This appendix includes a sample code used to analyse the HUH cohort's risk of Alzheimer's disease.

In [ ]:
```
library("dplyr")
library('lubridate')
library("ggplot2")
library("survminer")
library("ggpubr")
library("survival")
library("table1")
library("kableExtra")
```

Upload and organise the data

In [ ]:
```
# Upload data and calculate the avarege time between TCC diagnosis and BC
BCG_data <- read.csv(file = 'HUH_file.csv') %>%
    filter(!is.na(ageofbcg)) %>% filter(ageofbcg !=  AgeTCC)
mean(BCG_data$ageofbcg - BCG_data$AgeTCC)
```

In [ ]:
```
# main data for analysis
g2_data <- read.csv(file = 'HUH_file.csv') %>%
  mutate(end_age = DateAD - Birth) %>% #calculate terminal age of followup
  mutate(time = as.numeric(end_age - AgeTCC)) %>% #calculate time of followup
  filter(!(serialNumber %in% BCG_data$serialNumber)) %>% # remove bcg patient
  mutate(gender = ifelse(Male.1Female.0 == 1, "M", "F")) %>%
  mutate(is_BCG = ifelse(BCG.Yes.1.No.0 == 1, TRUE, FALSE)) %>%
  mutate(death = case_when(event == 1 ~ 1, event ==0 & DateAD == 2019 ~ 0,
                           event ==0 & DateAD < 2019 ~ 2)) %>%
  select(c(1,15,14,13,8,4,16,9,5))
colnames(g2_data) <- c("guid_tz", "is_BCG","gender", "time", "age_date_diagno
                       "AD_date")
```

Survival Analyses

In [ ]:
```
df_cox <- g2_data %>%
  filter(age_date_diagnosis >59) %>% # filter age at diagnosis
  filter(time>1) %>% # filter minimal followup time
  mutate(time2 = time*365)
  #filter(gender == 'M') # used for sub group analyses
  #filter(age_date_diagnosis <75) # used for sub group analyses

survObject <- survival::Surv(time = df_cox$time2, event = df_cox$event1)
fit1 <- survival::survfit(survObject ~ is_BCG, data = df_cox)

# KM, analysed through coxph without covarietes
simple_cox_all_data <- survival::coxph(survObject ~ is_BCG, data = df_cox,  m
summary(simple_cox_all_data)
```

```
# Cox
coxph_all_data <- survival::coxph(survObject ~ is_BCG  + age_date_diagnosis +
                                  data = df_cox,  method = 'breslow')
summary(coxph_all_data)

# KM plot
survminer::ggsurvplot(fit1, data = df_cox, title = "AD Hadassah All",
                      xlab = 'Time (Days)', conf.int = FALSE, legend.labs = c
                      ylim = c(0.65,1), pval = TRUE, pval.coord = c(0,0.7), r
                      palette = c("#E7B800", "#2E9FDF"),  ggtheme = theme_bw(
```

In [ ]:

```
# Use ZPH to test the Proportional Hazards Assumption of a Cox Regression
all_zph <- cox.zph(coxph_all_data, transform="km", global=TRUE)
all_zph
```

Competing risk analysis

In [ ]:

```
# Fine-Gray model
# Follows the code outline of: https://cran.r-project.org/web/packages/surviv

df_cox$time2 <- as.numeric(df_cox$time2)
df_cox$event <- factor(df_cox$event, 0:2, labels=c("censor", "AD", "death"))

dat1 <- finegray(Surv(df_cox$time2, df_cox$event) ~ ., data=df_cox,
                   etype="AD")
deathdat <- finegray(Surv(df_cox$time2, df_cox$event) ~ ., data=df_cox, etype

# The event curves of the multi-state model
pfit2 <- survfit(Surv(fgstart, fgstop, fgstatus) ~ is_BCG,
                 data=dat1, weight=fgwt)
# The death curves of the multi-state model
dfit2 <- survfit(Surv(fgstart, fgstop, fgstatus) ~ is_BCG,
                 data=deathdat, weight=fgwt)

# only for dat1
# extract the beta coefficient from here
fgfit1 <- coxph(Surv(fgstart, fgstop, fgstatus) ~ is_BCG+ age_date_diagnosis
                weight= fgwt)
fgfit2 <- coxph(Surv(fgstart, fgstop, fgstatus) ~ is_BCG+ age_date_diagnosis
                weight= fgwt)

round(rbind(PCM= coef(fgfit1), death=coef(fgfit2)), 3)
summary(fgfit1)
cox.zph(fgfit1, transform="km", global=TRUE)
```

Descriptive statistics example

In [ ]:

```
df_statistics <- df_cox %>%
  mutate(death_age = case_when((event == 'death') ~ (age_date_diagnosis+time)
                               (event != 'death') ~ as.numeric("NA"))) %>%
  #mutate(ptira_years = round(ptira_time/365)) %>%
  #mutate(death_age = age_date_diagnosis+ptira_years) %>%
  mutate(age_group = case_when((age_date_diagnosis < 65) ~ "60-64",
                               (age_date_diagnosis > 64 & age_date_diagnosis
                               (age_date_diagnosis > 69 & age_date_diagnosis
                               (age_date_diagnosis > 74 & age_date_diagnosis
                               (age_date_diagnosis > 79 & age_date_diagnosis
                               (age_date_diagnosis > 84 & age_date_diagnosis
                               (age_date_diagnosis >= 90) ~ "90+"))
```

```
# cosmetics
df_statistics$is_BCG <-
  factor(df_statistics$is_BCG, levels=c(FALSE,TRUE),
         labels=c("Non-BCG",
                  "BCG"))
df_statistics$event1 <-
  factor(df_statistics$event1, levels=c(0,1),
         labels=c("Non-AD",
                  "AD"))
df_statistics$gender <-
  factor(df_statistics$gender, levels=c("M","F"),
         labels=c("Male",
                  "Female"))

label(df_statistics$gender) <- "Gender"
label(df_statistics$age_date_diagnosis) <- "Age at TCC Diagnosis"
label(df_statistics$event)     <- "AD Diagnosis"
label(df_statistics$death_age) <- "Age of Death"
label(df_statistics$age_group) <- "Age Group"
label(df_statistics$time2) <- "Follow-Up Time"


units(df_statistics$age_date_diagnosis) <- "years"
units(df_statistics$death_age) <- "years"
units(df_statistics$age_group) <- "years"
units(df_statistics$time2) <- "days"

# Table1
table1(~ gender+ event+ age_date_diagnosis+ death_age + time2   | is_BCG, dat

# Table 2
table1(~ age_group | is_BCG*event1, data=df_statistics, topclass="Rtable1-zeb
```

For any questions please contact Danielle Klinger: dedeklinger[at]gmail.com

In [ ]:
