# Supplementary material for: Bladder Cancer Immunotherapy by BCG Is Associated with a Significantly Reduced Risk of Alzheimer’s Disease and Parkinson’s Disease
Source: Vaccines (Basel). 2021 May 11;9(5):491. doi: 10.3390/vaccines9050491 (PMC8151667; doi:10.3390/vaccines9050491)
Supplement: Supplementary file 1 [file vaccines-09-00491-s001.zip › vaccines-1206490 supp.pdf]

Bladder Cancer Immunotherapy by BCG is associated with a significantly reduced risk of Alzheimer's disease and Parkinson's disease

Danielle Klinger[1], Brian L. Hill[2], Noam Barda[3,4], Eran Halperin[2], Ofer N. Gofrit[5], Charles L. Greenblatt[6], Nadav Rappoport[7], Michal Linial[1]* and Hervé Bercovier[6]*

Supplemental Materials

Supplemental Inclusion-exclusion criteria

PD Specific Filtration: For analyses of PD risk, PD disease incidents were ascertained at least 365 days after the key date. Samples with less than one-year follow-up from the key date were excluded from the study. Any sample that was diagnosed with PD prior to BC diagnosis, was excluded from the cohort. In addition, samples with a stroke diagnosis were excluded.

Stroke Specific Filtration: For analyses of stroke risk, stroke incidents were ascertained at least 365 days after the key date. Samples with less than one-year follow-up from the key date were excluded from the study. Any sample that was diagnosed with stroke prior to BC diagnosis, was excluded from the cohort.

Diabetes Specific Filtration: For analyses of diabetes risk, diabetes incidents were ascertained at least 365 days after the key date. Samples with less than one-year follow-up from the key date were excluded from the study. Any sample that was diagnosed with diabetes prior to BC diagnosis, was excluded from the cohort.

Supplementary Figures

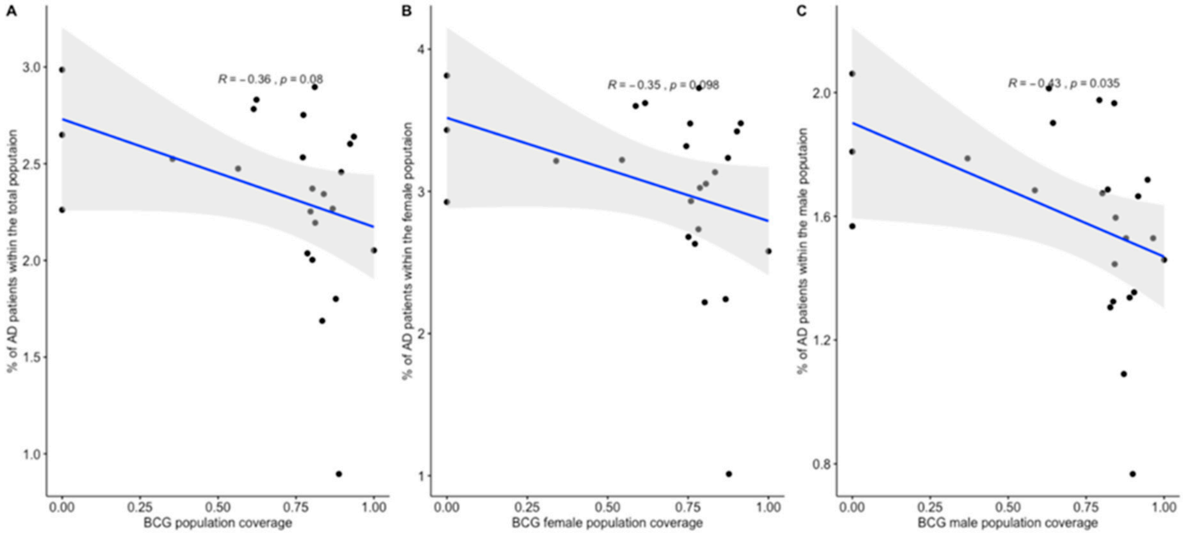

Figure S1. Statistical analysis of the correlation between the BCG population coverage and the percentage of AD patients within the total population. A. full cohort; B. female cohort; C. male cohort. Each black dot represents a European country; the grey area represents the 95% CI.

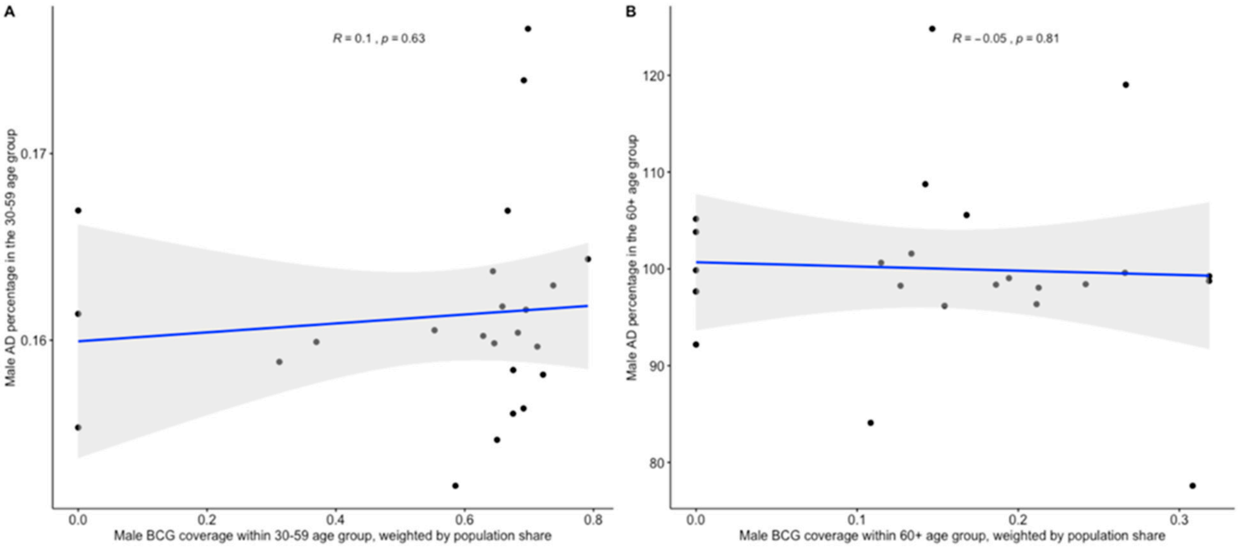

Figure S2. Statistical analysis of the correlation between the BCG population coverage and the percentage of AD patients within male population, in different age groups. A. 30-59 age group; B. >60 age group. Each black dot represents a country. Gray area represents 95% CI.

Supplementary Tables (S1-S9)

Table S1. **Characteristics of male and female patients in the CHS population.**

A

Male Cohort	Non-BCG (N=4217)	BCG (N=1341)	Overall (N=5558)
**AD Diagnosis**			
Non-AD	3960 (93.9%)	1277 (95.2%)	5237 (94.2%)
AD	257 (6.1%)	64 (4.8%)	321 (5.8%)
**Age at TCC Diagnosis (years)**			
Mean (SD)	73.9 (8.08)	72.8 (7.58)	73.6 (7.97)
Median [Min, Max]	74.0 [60.0, 104]	73.0 [60.0, 99.0]	73.0 [60.0, 104]
**Stage**			
Stage <T2	3805 (90.2%)	1212 (90.4%)	5017 (90.3%)
Stage ≥T2	412 (9.8%)	129 (9.6%)	541 (9.7%)
**Age of Death (years)**			
Mean (SD)	82.0 (8.08)	82.2 (7.68)	82.0 (8.00)
Median [Min, Max]	82.0 [61.0, 110]	83.0 [62.0, 103]	82.0 [61.0, 110]
Missing	1611 (38.2%)	681 (50.8%)	2292 (41.2%)
**Follow-Up Time (days)**			
Mean (SD)	2570 (1530)	2610 (1360)	2580 (1490)
Median [Min, Max]	2330 [366, 6570]	2470 [368, 6490]	2380 [366, 6570]

B

Female Cohort	Non-BCG (N=930)	BCG (N=237)	Overall (N=1167)
**AD Diagnosis**			
Non-AD	851 (91.5%)	226 (95.4%)	1077 (92.3%)
AD	79 (8.5%)	11 (4.6%)	90 (7.7%)
**Age at TCC Diagnosis (years)**			
Mean (SD)	74.3 (8.23)	73.2 (7.32)	74.1 (8.07)
Median [Min, Max]	74.0 [60.0, 98.0]	74.0 [60.0, 90.0]	74.0 [60.0, 98.0]
**Stage**			
Stage <T2	850 (91.4%)	203 (85.7%)	1053 (90.2%)
Stage ≥T2	80 (8.6%)	34 (14.3%)	114 (9.8%)
**Age of Death (years)**			
Mean (SD)	83.3 (8.19)	82.7 (7.49)	83.2 (8.08)
Median [Min, Max]	84.0 [63.0, 101]	83.0 [63.0, 96.0]	84.0 [63.0, 101]
Missing	425 (45.7%)	143 (60.3%)	568 (48.7%)
**Follow-Up Time (days)**			
Mean (SD)	2790 (1570)	2770 (1400)	2790 (1540)
Median [Min, Max]	2690 [369, 6470]	2760 [379, 6390]	2700 [369, 6470]

(**A**) Male population. (**B**), Female population. Non-BCG are bladder cancer patients not treated with BCG post filtration (control group) as described in the inclusion-exclusion paragraph; BCG are bladder cancer patients treated by BCG instillation after filtration as described in the inclusion-exclusion paragraph. Abbreviations: AD, Alzheimer's disease; TCC, Transitional cell cancer; Stage, bladder cancer stage at diagnosis.

TableS 2. CHS AD and age distribution of patients (male / female), treated or not with BCG.

A

Full Cohort	Non-BCG		BCG		Overall	
	Non-AD (N=4811)	AD (N=336)	Non-AD (N=1503)	AD (N=75)	Non-AD (N=6314)	AD (N=411)
**Age Group (years)**						
60-64	751 (15.6%)	11 (3.3%)	255 (17.0%)	0 (0%)	1006 (15.9%)	11 (2.7%)
65-69	896 (18.6%)	25 (7.4%)	291 (19.4%)	11 (14.7%)	1187 (18.8%)	36 (8.8%)
70-74	962 (20.0%)	63 (18.8%)	342 (22.8%)	18 (24.0%)	1304 (20.7%)	81 (19.7%)
75-79	1002 (20.8%)	104 (31.0%)	309 (20.6%)	21 (28.0%)	1311 (20.8%)	125 (30.4%)
80-84	702 (14.6%)	91 (27.1%)	212 (14.1%)	15 (20.0%)	914 (14.5%)	106 (25.8%)
85-89	351 (7.3%)	33 (9.8%)	80 (5.3%)	9 (12.0%)	431 (6.8%)	42 (10.2%)
90+	147 (3.1%)	9 (2.7%)	14 (0.9%)	1 (1.3%)	161 (2.5%)	10 (2.4%)

B

Male Cohort	Non-BCG		BCG		Overall	
	Non-AD (N=3960)	AD (N=257)	Non-AD (N=1277)	AD (N=64)	Non-AD (N=5237)	AD (N=321)
**Age Group (years)**						
60-64	618 (15.6%)	11 (4.3%)	221 (17.3%)	0 (0%)	839 (16.0%)	11 (3.4%)
65-69	742 (18.7%)	16 (6.2%)	249 (19.5%)	11 (17.2%)	991 (18.9%)	27 (8.4%)
70-74	809 (20.4%)	46 (17.9%)	297 (23.3%)	13 (20.3%)	1106 (21.1%)	59 (18.4%)
75-79	809 (20.4%)	86 (33.5%)	251 (19.7%)	17 (26.6%)	1060 (20.2%)	103 (32.1%)
80-84	585 (14.8%)	66 (25.7%)	176 (13.8%)	15 (23.4%)	761 (14.5%)	81 (25.2%)
85-89	278 (7.0%)	26 (10.1%)	70 (5.5%)	7 (10.9%)	348 (6.6%)	33 (10.3%)
90+	119 (3.0%)	6 (2.3%)	13 (1.0%)	1 (1.6%)	132 (2.5%)	7 (2.2%)

C

Female Cohort	Non-BCG		BCG		Overall	
	Non-AD (N=851)	AD (N=79)	Non-AD (N=226)	AD (N=11)	Non-AD (N=1077)	AD (N=90)
**Age Group (years)**						
60-64	133 (15.6%)	0 (0%)	34 (15.0%)	0 (0%)	167 (15.5%)	0 (0%)
65-69	154 (18.1%)	9 (11.4%)	42 (18.6%)	0 (0%)	196 (18.2%)	9 (10.0%)
70-74	153 (18.0%)	17 (21.5%)	45 (19.9%)	5 (45.5%)	198 (18.4%)	22 (24.4%)
75-79	193 (22.7%)	18 (22.8%)	58 (25.7%)	4 (36.4%)	251 (23.3%)	22 (24.4%)
80-84	117 (13.7%)	25 (31.6%)	36 (15.9%)	0 (0%)	153 (14.2%)	25 (27.8%)
85-89	73 (8.6%)	7 (8.9%)	10 (4.4%)	2 (18.2%)	83 (7.7%)	9 (10.0%)
90+	28 (3.3%)	3 (3.8%)	1 (0.4%)	0 (0%)	29 (2.7%)	3 (3.3%)

(**A**) Full CHS cohort. (**B**) CHS Male population. (C) CHS Female population. Non-BCG are bladder cancer patients not treated with BCG post filtration (control group) as described in the inclusion-exclusion paragraph; BCG are bladder cancer patients treated by BCG instillation after filtration as described in the inclusion-exclusion paragraph. AD are patients diagnosed with AD, Non-AD are patients not diagnosed with AD throughout the analysis. Abbreviations: AD, Alzheimer's disease; Age group, bladder cancer age at diagnosis.

Table S3. AD and age distribution of patients, treated or not with BCG, **HUH Population.**

	Non-BCG		BCG		Overall	
	Non-AD (N=275)	AD (N=17)	Non-AD (N=395)	AD (N=13)	Non-AD (N=670)	AD (N=30)
**Age Group (years)**						
60-64	31 (11.3%)	2 (11.8%)	54 (13.7%)	0 (0%)	85 (12.7%)	2 (6.7%)
65-69	61 (22.2%)	1 (5.9%)	82 (20.8%)	2 (15.4%)	143 (21.3%)	3 (10.0%)
70-74	47 (17.1%)	5 (29.4%)	88 (22.3%)	2 (15.4%)	135 (20.1%)	7 (23.3%)
75-79	56 (20.4%)	2 (11.8%)	72 (18.2%)	3 (23.1%)	128 (19.1%)	5 (16.7%)
80-84	45 (16.4%)	5 (29.4%)	54 (13.7%)	2 (15.4%)	99 (14.8%)	7 (23.3%)
85-89	27 (9.8%)	2 (11.8%)	29 (7.3%)	4 (30.8%)	56 (8.4%)	6 (20.0%)
90+	8 (2.9%)	0 (0%)	16 (4.1%)	0 (0%)	24 (3.6%)	0 (0%)

Legend Table S3. AD are patients diagnosed with AD, Non-AD are patients not diagnosed with AD throughout the analysis. Abbreviations: AD, Alzheimer's disease; Age group, bladder cancer age at diagnosis;.Non-BCG are bladder cancer patients not treated with BCG post filtration (control group) as described in the inclusion-exclusion paragraph; BCG are bladder cancer patients treated by BCG instillation after filtration as described in the inclusion-exclusion paragraph.

Table S4. Risk of AD in the CHS Cohort by gender using Kaplan-Meier (KM), Cox Proportional Hazards Regression (Cox) and Competing Risk Analyses (CR).

Disease Risk Assesment

Analysis	HR (95% CI)	P value
**CHS AD Female**		
KM	0.572 (0.304–1.075)	0.082
Cox	0.588 (0.313–1.106)	0.100
CR	0.639 (0.340–1.202)	0.165
**CHS AD Male**		
KM	0.779 (0.592–1.024)	0.074
Cox	0.836 (0.635–1.100)	0.200
CR	0.886 (0.673–1.166)	0.387

Legend Table S4. CHS AD Female, includes female patients age ≥60 diagnosed with AD; CHS AD Male, includes male patients age ≥60 diagnosed with AD; CHS PD, includes patients age ≥60 diagnosed with PD. Abbreviations: HR, Hazard Ratio; KM, The HR derived from the use of an unadjusted Cox regression analysis for Kaplan Meier (KM) survival analysis; Cox, Cox Proportional Hazards Regression; CR, Fine-Gray Competing Risk model; AD, Alzheimer's' Disease; PD, Parkinson Disease; CHS, Clalit Health services; HUH, Hadassah University Hospitals. HR of 1.00 indicates lack of association; greater than 1.00, an increased risk of death; and less than 1.00, a decreased risk of death. P-values marked with * are <0.001. Analyses were conducted using R.

Table S5. Characteristics of patients in the UCLAH population.

	Non-BCG (N=2138)	BCG (N=132)	Overall (N=2270)
Gender			
Male	1576 (73.7%)	104 (78.8%)	1680 (74.0%)
Female	562 (26.3%)	28 (21.2%)	590 (26.0%)
AD Diagnosis			
Non-AD	2059 (96.3%)	132 (100%)	2191 (96.5%)
AD	79 (3.7%)	0 (0%)	79 (3.5%)
Age at TCC Diagnosis (years)			
Mean (SD)	70.4 (12.2)	69.1 (12.3)	70.3 (12.2)
Median [Min, Max]	71.0 [14.0, 90.0]	69.0 [26.0, 90.0]	71.0 [14.0, 90.0]
Age of Death (years)			
Mean (SD)	76.4 (12.7)	76.5 (14.4)	76.4 (12.8)
Median [Min, Max]	78.0 [25.0, 102]	78.5 [49.0, 92.0]	78.0 [25.0, 102]
Missing	1973 (92.3%)	124 (93.9%)	2097 (92.4%)
Follow-Up Time (days)			
Mean (SD)	1480 (886)	1220 (502)	1470 (871)
Median [Min, Max]	1310 [366, 7290]	1180 [375, 2680]	1290 [366, 7290]

Legend Table S5. Non-BCG are bladder cancer patients not treated with BCG post filtration (control group) as described in the inclusion-exclusion paragraph; BCG are bladder cancer patients treated by BCG instillation after filtration as described in the inclusion-exclusion paragraph. Abbreviations: AD, Alzheimer's disease; TCC, Transitional cell cancer; Stage, bladder cancer stage at diagnosis;

Table S6. AD and age distribution of patients treated or not with BCG, **UCLAH population.**

	Non-BCG		BCG	Overall	
	Non-AD (N=2059)	AD (N=79)	Non-AD (N=132)	Non-AD (N=2191)	AD (N=79)
Age Group (years)					
60-64	592 (28.8%)	2 (2.5%)	42 (31.8%)	634 (28.9%)	2 (2.5%)
65-69	337 (16.4%)	4 (5.1%)	26 (19.7%)	363 (16.6%)	4 (5.1%)
70-74	380 (18.5%)	5 (6.3%)	22 (16.7%)	402 (18.3%)	5 (6.3%)
75-79	298 (14.5%)	16 (20.3%)	14 (10.6%)	312 (14.2%)	16 (20.3%)
80-84	215 (10.4%)	24 (30.4%)	12 (9.1%)	227 (10.4%)	24 (30.4%)
85-89	168 (8.2%)	18 (22.8%)	13 (9.8%)	181 (8.3%)	18 (22.8%)
90+	69 (3.4%)	10 (12.7%)	3 (2.3%)	72 (3.3%)	10 (12.7%)

Legend Table S6. Non-BCG are bladder cancer patients not treated with BCG post filtration (control group) as described in the inclusion-exclusion paragraph; BCG are bladder cancer patients treated by BCG instillation after filtration as described in the inclusion-exclusion paragraph. AD are patients diagnosed with AD, Non-AD are patients not diagnosed with AD throughout the analysis. Abbreviations: AD, Alzheimer's disease; Age group, bladder cancer age at diagnosis.

Table S7. **Characteristics of PD patients in the CHS Population.**

Full Cohort	Non-BCG (N=5097)	BCG (N=1669)	Overall (N=6766)
**Gender**			
Male	4157 (81.6%)	1409 (84.4%)	5566 (82.3%)
Female	940 (18.4%)	260 (15.6%)	1200 (17.7%)
**PD Diagnosis**			
Non-PD	4944 (97.0%)	1636 (98.0%)	6580 (97.3%)
PD	153 (3.0%)	33 (2.0%)	186 (2.7%)
**Age at TCC Diagnosis (years)**			
Mean (SD)	74.0 (8.14)	72.8 (7.59)	73.7 (8.02)
Median [Min, Max]	74.0 [60.0, 104]	73.0 [60.0, 99.0]	74.0 [60.0, 104]
**Stage**			
Stage T2<	4608 (90.4%)	1500 (89.9%)	6108 (90.3%)
Stage >=T2	489 (9.6%)	169 (10.1%)	658 (9.7%)
**Age of Death (years)**			
Mean (SD)	82.2 (8.12)	82.2 (7.65)	82.2 (8.03)
Median [Min, Max]	83.0 [61.0, 110]	83.0 [62.0, 104]	83.0 [61.0, 110]
Missing	2017 (39.6%)	885 (53.0%)	2902 (42.9%)
**Follow-Up Time (days)**			
Mean (SD)	2630 (1550)	2590 (1370)	2620 (1500)
Median [Min, Max]	2410 [366, 6570]	2460 [368, 6490]	2420 [366, 6570]

Legend Table S7. Non-BCG are bladder cancer patients not treated with BCG post filtration (control group) as described in the inclusion-exclusion paragraph; BCG are bladder cancer patients treated by BCG instillation after filtration as described in the inclusion-exclusion paragraph.Abbreviations: PD, Parkinson disease; TCC, Transitional cell cancer; Stage, bladder cancer stage at diagnosis;.

Table S8. CHS PD and age distribution of patients, treated or not with BCG.

Full Cohort	Non-BCG		BCG		Overall	
	Non-PD (N=4944)	PD (N=153)	Non-PD (N=1636)	PD (N=33)	Non-PD (N=6580)	PD (N=186)
**Age Group (years)**						
60-64	747 (15.1%)	12 (7.8%)	273 (16.7%)	0 (0%)	1020 (15.5%)	12 (6.5%)
65-69	890 (18.0%)	21 (13.7%)	311 (19.0%)	7 (21.2%)	1201 (18.3%)	28 (15.1%)
70-74	983 (19.9%)	32 (20.9%)	372 (22.7%)	7 (21.2%)	1355 (20.6%)	39 (21.0%)
75-79	1036 (21.0%)	51 (33.3%)	335 (20.5%)	14 (42.4%)	1371 (20.8%)	65 (34.9%)
80-84	759 (15.4%)	19 (12.4%)	235 (14.4%)	3 (9.1%)	994 (15.1%)	22 (11.8%)
85-89	375 (7.6%)	15 (9.8%)	91 (5.6%)	2 (6.1%)	466 (7.1%)	17 (9.1%)
90+	154 (3.1%)	3 (2.0%)	19 (1.2%)	0 (0%)	173 (2.6%)	3 (1.6%)

Legend Table S8. Non-BCG are bladder cancer patients not treated with BCG post filtration (control group) as described in the inclusion-exclusion paragraph; BCG are bladder cancer patients treated by BCG instillation after filtration as described in the inclusion-exclusion paragraph. PD are patients diagnosed with PD, Non-PD are patients not diagnosed with PD throughout the analysis. Abbreviations: PD, Parkinson disease; Age group, bladder cancer age at diagnosis.

Table S9. Risk of Stroke and Type-2 Diabetes in the CHS Cohort. By Kaplan-Meier and Cox Proportional Hazards Regression.

Risk Assesment, Stroke and T2D

Analysis	HR (95% CI)	P value
**CHS Stroke Full**		
KM	1.048 (0.846–1.229)	0.667
Cox	1.068 (0.861–1.326)	0.547
**CHS Stroke M**		
KM	1.120 (0.894–1.412)	0.319
Cox	1.146 (0.911–1.441)	0.244
**CHS Stroke F**		
KM	0.616 (0.315–1.206)	0.158
Cox	0.646 (0.330–1.266)	0.203
**CHS T2D**		
KM	0.710 (0.429–1.490)	0.482
Cox	0.890 (0.479–1.679)	0.732

Table S9. Risk of Stroke and Type-2 Diabetes in the CHS Cohort. By Kaplan-Meier and Cox Proportional Hazards Regression. CHS Stroke full, includes all cohort patients age ≥60 diagnosed with Stroke; CHS Stroke M, includes male patients age ≥60 diagnosed with Stroke; CHS Stroke F, includes female patients age ≥60 diagnosed with Stroke; CHS DT2, includes patients age ≥60 diagnosed with Type 2 Diabetes. Abbreviations: HR, Hazard Ratio; KM, HR derived from the use of an unadjusted cox regression analysis [HR(s)]; Cox, Cox Proportional Hazards Regression; CHS, Clalit Health services. HR of 1.00 indicates lack of association; greater than 1.00, an increased risk of death; and less than 1.00, a decreased risk of death. Analyses were conducted using R.

Technical Appendix

A technical appendix added in R format. The technical appendix includes a sample code used to analyze the HUH cohort's risk of Alzheimer's disease. This code is representative of the analyses and filtrations conducted in the CHS, HUH and UCLAH cohorts for any type of disease outcome.
